# Severe Adverse Toxic Effects of Low-Dose Methotrexate Treatment on an Ectopic Pregnancy Patient With Methylenetetrahydrofolate Reductase Mutations: A Case Report

**DOI:** 10.3389/fmed.2021.738315

**Published:** 2021-11-18

**Authors:** Huan Yu, Wenhui Wang, Haiyan Liang, Kun Wang, Bin Ling

**Affiliations:** ^1^Department of Obstetrics and Gynaecology, China-Japan Friendship Hospital, Beijing, China; ^2^Graduate School of Peking Union Medical College, Chinese Academy of Medical Sciences, Beijing, China

**Keywords:** methotrexate, ectopic pregnancy, methylenetetrahydrofolate reductase (*MTHFR*) gene, myelosuppression, treatment

## Abstract

**Background:** Low-dose methylenetetrahydrofolate (LD-MTX) has been widely used for the treatment of the ectopic pregnancy (EP) for many decades, and related severe adverse toxic effects are rare. Current studies have shown that the polymorphisms of methylenetetrahydrofolate reductase (*MTHFR*) gene can decrease the MTX clearance, leading to the metabolite accumulation. However, there is a lack of literature report on an *MTHFR* gene polymorphism associated with adverse toxic effects resulting from the use of LD-MTX in an EP.

**Case Presentation:** We report a rare case of a 38-year-old female who developed persistent fever, grade IV myelosuppression, skin lesions, mucositis, and liver injury after single dose of LDMTX to treat EP. The personalized genetic testing showed that *MTHFR* TT (677C>T) and *MTHFR* AA (1298A>C) were detected. Gradually, the symptoms improved after calcium leucovorin (CF) rescue, continuous renal replacement therapy (CRRT), promoting blood system regeneration, and multiple supportive treatments.

**Conclusion:** This is the first report on the serious adverse toxic effects of LD-MTX on an EP patient with *MTHFR* mutations. We aim to alert obstetricians and gynecologists to this rare condition. The unexpected life-threatening toxicity with LD-MTX should be highly considered and recognized early. In particular, some easily overlooked gastrointestinal, skin, and mucosal symptoms occur earlier than severe myelosuppression. When toxic effects are suspected, detecting the polymorphisms of an *MTHFR* gene and monitoring MTX concentration in blood could assist us to formulate individualized and active treatments.

## Introduction

Methotrexate (MTX) is an antagonist of folic acid, which inhibits dihydrofolate reductase (DHFR) and prevents the proliferation of trophoblasts (1). MTX was first reported to treat ectopic pregnancy (EP) in 1982 (2). To date, low-dose MTX (LD-MTX) has been widely used for the treatment of the unruptured EP (UEP) to avoid the necessity of undergoing surgery and preserve the integrity of the fallopian tube (3, 4). Furthermore, MTX is highly toxic to rapidly replicating tissues, and it may cause hematologic, gastrointestinal (hepatic, nausea, etc.), and mucocutaneous adverse effects (5–7). However, in the case of administration of LD-MTX, its toxic effects are generally mild and self-limiting (8). LD-MTX has been used to treat EP for a long time, but only a limited number of its adverse effects have been reported, especially in the single-dose MTX regimen that seems to be a relatively safe treatment (9–13). Nonetheless, further research needs to be conducted to indicate the possible adverse effects of LD-MTX on EP patients who have already received it (9, 10).

A number of previous studies have shown that severe adverse toxic effects of LD-MTX are associated with polymorphisms of methylenetetrahydrofolate reductase (*MTHFR*) gene, such as *MTHFR* 677TT and 1298AA (8, 14–16), as the polymorphisms of *MTHFR* gene can decrease the MTX clearance, leading to the metabolite accumulation (15, 17). For the patients who had serious adverse toxic effects of LD-MTX, it is essential to consider genetic abnormalities. Herein, we reported a rare case of EP with *MTHFR* gene mutations who developed serious adverse toxic effects of LD-MTX.

## Case Presentation

A 38-year-old woman, gravida 3 para 1 abortion 1, was admitted to the Department of Obstetrics and Gynecology of the China-Japan Friendship Hospital (Beijing, China) on October 5, 2019, with complaints of 7 weeks amenorrhea, cornual pregnancy treated with MTX for 5 days and fever (38.5°C) for 2 days. About a week earlier, the patient was presented in a local hospital, and was diagnosed with cornual pregnancy, vaginal bleeding, the elevated beta-human chorionic gonadotropin (β-hCG) level (987–1,959 mIU/L) and ultrasound findings that a mixed echogenic mass of 13.4 × 7.9 mm in the right cornu uteri. Then, she intramuscularly (IM) received the routine LD-MTX (75 mg) for EP on October 1, 2019. After 1 day of LD-MTX administration (“day 1” was abbreviated as “D1”), nausea, vomiting, and diarrhea appeared, while no special treatment was given. Next, other symptoms, such as fever (38.5°C), sore throat, and left back pain, appeared (D2). On admission (D4), a few maculopapular rashes on the left neck were observed, the β-hCG level was 8,279 mIU/L, and ultrasound showed the mixed echogenic mass in the right cornua uteri was 20 × 16 mm. CT of chest ruled out pulmonary infection and the patient denied the history of allergic diseases. So, she was diagnosed cornual pregnancy (an uncommon form of EP) that failed LD-MTX treatment. Whether the fever and skin rashes were due to LD-MTX or other causes, it had to be further investigated for a definitive answer.

During the next 3 days (“D5–D7”), the skin rashes of the patient progressed to small blisters, and covered ~80% of her body surface, excluding the palms and soles. Areas of desquamation were identified, while Nikolsky's sign, which is typically present in the cases of toxic epidermal necrolysis (TEN), was negative. Oral mucositis was gradually aggravated. Oral mucositis caused difficulty in the oral intake and swallowing. The perianal mucositis and fecal occult blood appeared on D8. In addition, a persistent fever was found until 20 days after administration of LD-MTX. Physical examination further revealed purulent secretions in the bilateral tonsillar fossa, ear swelling, percussion pain in both the kidneys, especially in the left kidney, and a mild tenderness in the right adnexal area.

**Figure 1:**
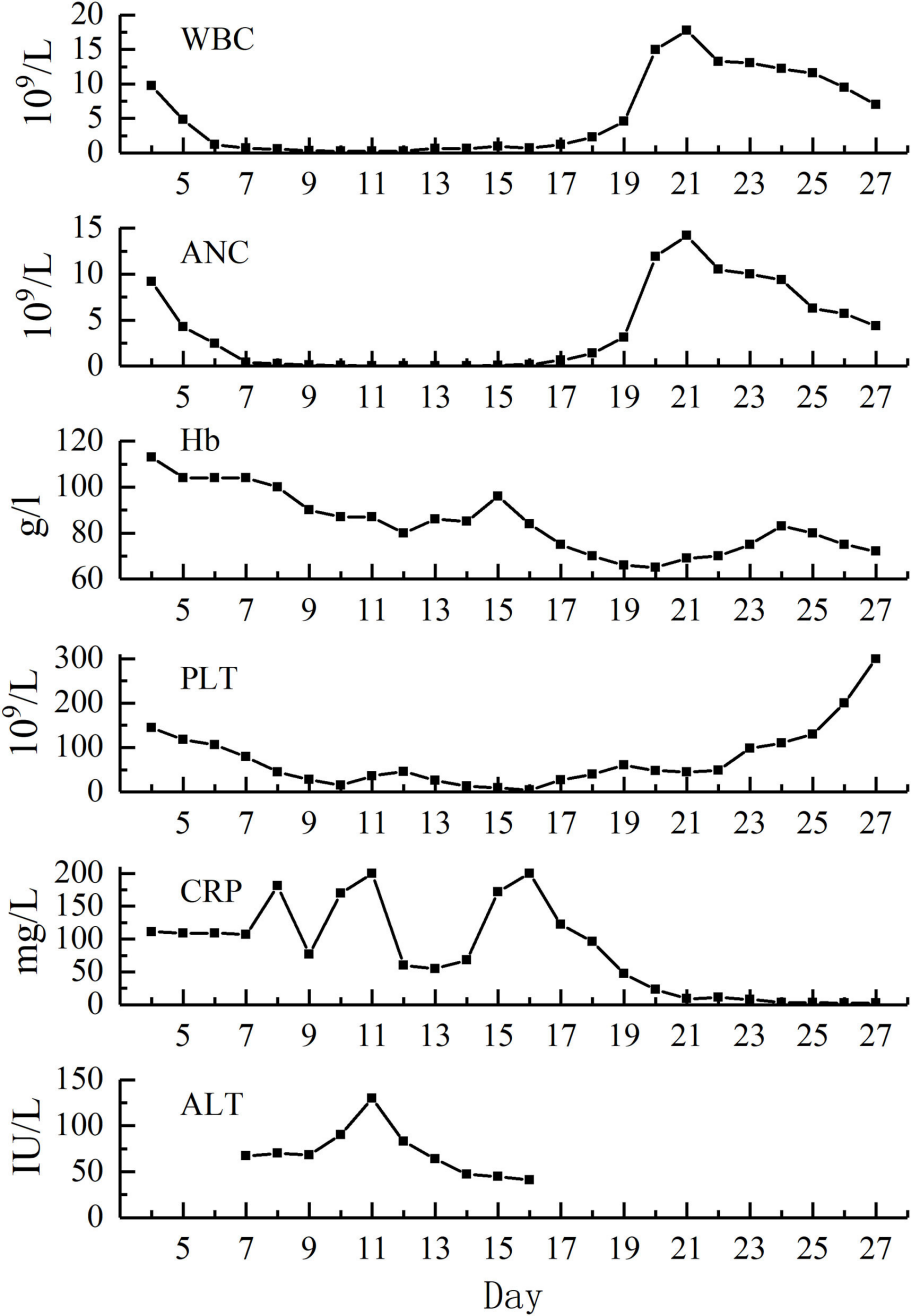
Hematological indices of the patient from admission to D27.

The testing of peripheral blood sample of the patient on admission performed in our hospital revealed the following outcomes: hemoglobin (110 g/L), absolute neutrophils count (ANC, 9.18 × 10^9^/L), platelet count (144 × 10^9^/L), C-reactive protein (CRP, 111 mg/L), alanine aminotransferase (ALT, 13 IU/L), total bilirubin (TBIL, 20.95 μM/L). Laboratory examinations carried out on the next days showed severe neutropenia (nadir dropped to zero, D11–D13), thrombocytopenia (nadir was 4 × 10^9^/L, D16), decreased hemoglobin (nadir was 65 g/L, D20), elevated CRP (the highest value was 200 mg/L, D11 and D16), and increased ALT and TBIL (the highest values were 130 IU/L and 34.37 uM/L, respectively, D11). The renal function of the patient was normal as well (Figure 1; Table 1).

**Table 1:** Laboratory data in relation to LD-MTX administration.

**Date**	Day^*^	**WBC (10^**9**^/L)**	**ANC (10^**9**^/L)**	**Hb (g/L)**	**PLT (10^**9**^/L)**	**CRP (mg/L)**	**ALT (IU/L)**	**TBIL (uM/L)**	**β-HCG (mM/L)**	**MTX (uM/L)**
10–5	D4	9.7	9.18	113	144	111	–	–	8279	–
10–6	D5	4.8	4.26	104	118	–	–	20.95	5596	–
10–7	D6	1.2	2.45	104	106	109	–			
10–8	D7	0.66	0.38	104	79	107	67	–	3946	–
10–9	D8	0.53	0.26	100	45	181	70	29.18	–	0.04
10–10	D9	0.31	0.09	90	28	77	68	27.59	1238	–
10–11	D10	0.22	0.04	87	15	170	90	31.23	–	–
10–12	D11	0.23	0.00	87	36	200	130	34.37	342.11	0.013
10–13	D12	0.24	0.00	80	46	60	83	19.12	152.41	–
10–14	D13	0.63	0.00	86	26	55	64	20.66	64.90	0.01
10–15	D14	0.60	0.02	85	13	68	47	21.85	21.51	0.01
10–16	D15	0.94	0.07	96	9	172	45	19.57	–	<0.018
10–17	D16	0.66	0.16	84	4	>200	41	13.54	–	<0.018
10–18	D17	1.2	0.62	75	27	122	–	–	-	–
10–19	D18	2.27	1.39	70	40	96	–	–	–	–
10–20	D19	4.57	3.12	66	60	47	–	–	–	–
10–21	D20	14.95	11.93	65	48	23	–	–	2.06	–
10–22	D21	17.77	14.19	69	45	9	–	–	–	–
10–23	D22	13.25	10.5	70	49	11	–	–	–	–
10–24	D23	13.05	10	75	98	8	–	–	–	–
10–25	D24	12.21	9.38	83	110	3.5	–	–	–	–
10–26	D25	11.57	6.28	80	130	3	–	–	–	–
10–27	D26	9.46	5.7	75	200	2.8	–	–	–	–
10–28	D27	7	4.36	72	341	<2.5	–	–	–	–

*WBC, white blood cell; ANC, absolute neutrophils count; Hb, hemoglobin; PLT, platelet count; CRP, C-reactive protein; ALT, alanine aminotransferase; TBIL, total bilirubin; β-HCG, beta-human chorionic gonadotropin; MTX, methotrexate*.

* *The day of MTX initiation was D0*.

Although LD-MTX was given, therapeutic drug monitoring showed that the serum MTX concentrations were 0.04 μM/L (D7) and 0.013 μM/L (D11), which were significantly higher than the normal level. In addition, the personalized genetic testing of MTX showed that *MTHFR* TT (677C>T) and *MTHFR* AA (1298A>C) were detected, indicating a 75% decrease in the *MTHFR* activity. The administration of folic acid was seriously prohibited, which resulted in the decrease of MTX clearance, as well as a relatively high risk of hematotoxicity, hepatotoxicity, and mucosal toxicity after receiving LD-MTX.

Being neutropenic and febrile, the patient was treated simultaneously with the prophylactic broad-spectrum antibiotics since D4. Due to the grade IV neutropenia, prophylactic broad-spectrum antibiotics were switched to ertapenem [1 g, q8h, intravenous (IV)], vancomycin (1 g, qd, IV), and fluconazole sodium (200 mg, qd, IV). She was simultaneously treated with granulocyte colony-stimulating factor (G-CSF, 300 μg, bid, IH) and thrombopoietin (TPO, 15,000 units/day, IH). MTX retention was treated by initiating calcium leucovorin (CF) rescue, which was started since D8 (20 mg, q6h, IV), and the dose was elevated on D11 (100 mg, q6h, IV) due to the abnormal MTX concentration. Continuous renal replacement therapy (CRRT) was performed four times from D7 to D13 after LD-MTX administration, with 2,000 ml plasma each time. Supportive measures were required, such as protective isolation, IV fluid, urine alkalinization, oral washes and perineum rinsing, and transfusion of 4 units of the platelet.

**Figure 2:**
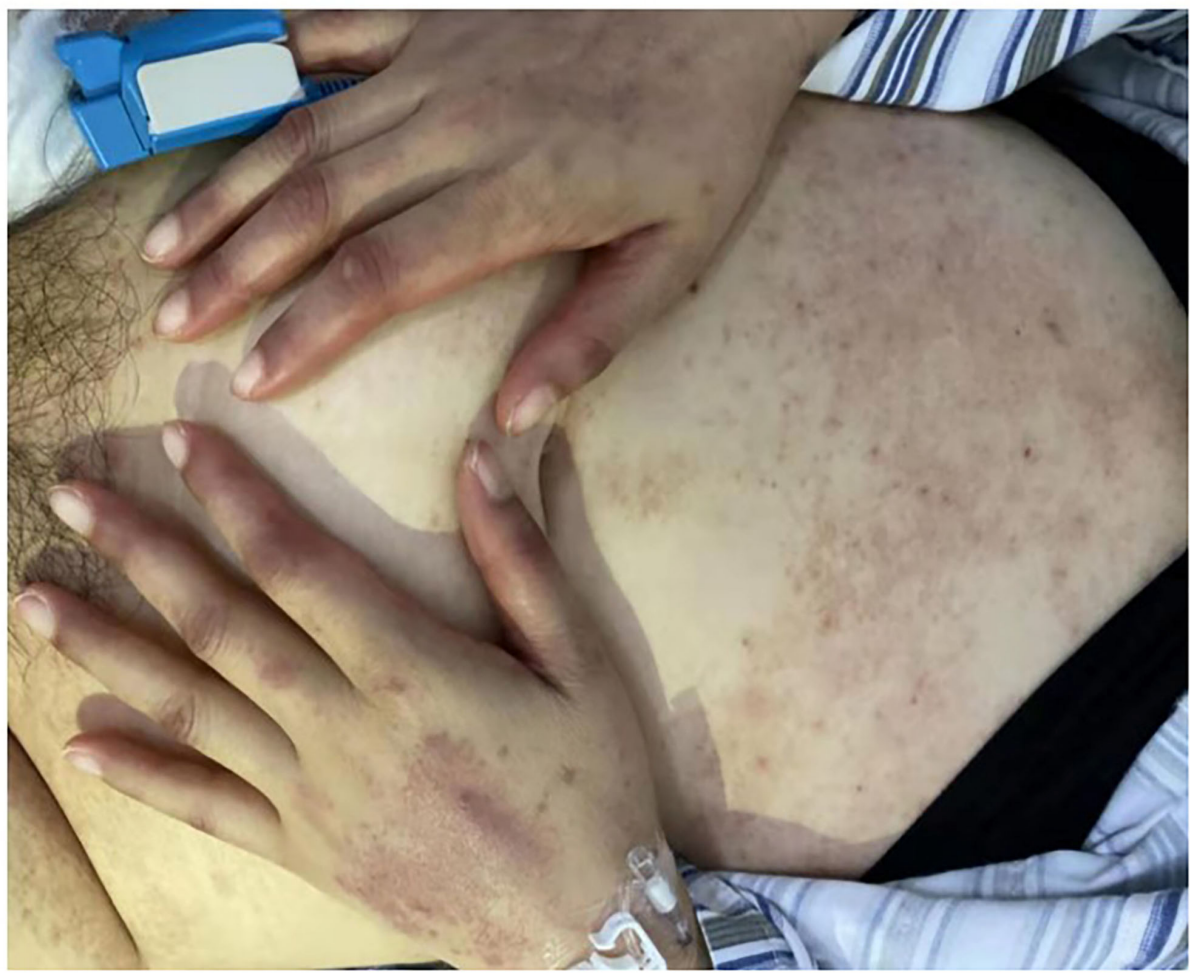
Red rashes over the abdomen and the back of hands after admission (D7).

On D17, the peripheral blood counts began to return to normal gradually. On D19, the G-CSF treatment was discontinued, and hemoglobin level and platelet count reached 70 g/L and 60 × 10^9^/L, respectively. On D20, the β-HCG level descended to the normal level. On D27, the mucositis and skin rashes had improved significantly, the patient reported no feeling of discomfort, and was discharged from the hospital (Figure 2). The β-HCG level was monitored until it turned negative more than twice; peripheral blood counts, and the function of the liver and kidney were monitored regularly; the transvaginal ultrasound was carried out again and showed no obvious abnormalities. To date, we have followed up her for 20 months, and her general condition is satisfactory.

## Discussion

Ectopic pregnancy is a common gynecologic emergency with an incidence rate of 1–3% among pregnant women (4, 18). At present, LD-MTX is the most effective therapeutic strategy for the EP patients (3, 18). MTX is a folic acid analog that remarkably and persistently inhibits DHFR, and consequently decreases the production of thymidylate and DNA. LD-MTX can interfere with the DNA synthesis and inhibit proliferation of trophoblasts, preventing embryonic development, and it may lead to absorption (1, 16). To date, only eight cases, including the present one, of single-dose LD-MTX protocols that induced serious adverse toxic effects on patients with EP have been reported (Table 2) (10, 19–24).

**Table 2:** Single LD-MTX induced severe adverse toxic effects in patients with EP.

**References**	**Case**	**Age (year)**	**MTX dose**	**Risk factors**	Myelosuppression (onset^*^) Nadir of blood counts	Initial symptoms (onset^*^)	**Other major adverse toxic effects**	**Treatment**	**Blood MTX concen-tration(μM/L)**	**MTFHR gene polymorphysm**	**Outcome**
Shafie et al. (19)	1	30	80 mg, i.m.	No	III° (D7) WBC: 1 × 109/L, D9 Hb: 66 g/L, D13 PLT: 10 × 109/L, D9	Pustular rashes, alopecia, hyperpigmentation, nausea, vomiting, mucositis, and raised Cr level (D1)	Fever, septic shock, and loss of consciousness	CF rescue, 20 mg, q6h	0.36, D3 0.07, D7 0.03, D12	No report	Recovery began on D16
Shao et al. (20)	2	27	50 mg, i.m.	No	III° (D6) WBC: 0.7 × 109/L, D6 Hb: 80 g/L, D10 PLT: 11 × 109/L, D12	Mucositis, nausea and vomiting (D4)	Fever and rash	CF rescue, 120 mg, qd	<0.12, D7	No report	Recovery began on D16
Gaïes et al. (21)	3	32	75 mg, i.m.	No	IV°(D7) WBC: 0.3 × 109/L, D7+ Hb: 77 g/L, D7+	Mucositis, odynophagia and dysphagia(D2)	Fever, skin lesions, hyperpigmentation, diarrhea, liver injury and septic shock	CF rescue, 60 mg, qd	0.05, D11 0.03, D13.	No report	Died
Willner et al. (10)	4	21	100 mg, i.v.	Hemodialysis dependent	III° (D4) WBC: 0.73 × 109/L, D13 Hb: 79.9 g/L, D10 PLT: 9.9 × 109/L, D15	Fever, oral thrush, mucositis, pruritis, and rash (D2)	–	CF rescue, 30 mg, q6h	0.12, D4 0.13, D5 0.07, D8 Undetectable^**^, D15	No report	Recovery began on D15
Kelly et al. (22)	5	Young	50 mg/m^2^ i.v.	Hemodialysis dependent	IV° (D8) WBC: 0.4 × 109/L, D14 Hb: 77 g/L, D10 PLT: 10 × 109/L, D 25	Nausea, vomiting, and oral thrush(D5)	Fever, mild delirium, diarrhea, TEN, liver injury, ARDS, and profound bowel ischemi	CF rescue, No report	0.11, D8 0.10, D10 0.02, D16	No report	Died
Isaacs et al. (23)	6	23	50 mg/m^2^ i.m.	No	IV° (D4) ANC^*^: 0.3 × 109/L, D11 Hb: 67 g/L, D11 PLT: 17 × 109/L, D11	Mucositis, pruritic rash, nausea, and vomiting (D3)	Fever	CF rescue, No report	No report	No report	Recovery began on D14
Present case	7	38	75 mg i.m.	No	IV° (D4) WBC: 0.24 × 109/L, D13 Hb: 65 g/L, D21 PLT: 4 × 109/L, D17	Nausea, vomiting and diarrhea (D1)	Fever, skin lesions, mucositis, and liver injury	CF rescue, 100 mg, q6h CRRT	0.04, D8 0.013, D11 0.001, D13 Undetectable^*^, D15	MTHFR (677C>T) TT and MTHFR (1298A>C) AA	Recovery began on D17

*MTX, methotrexate; CF, calcium leucovorin; MTHFR, methylenetetrahydrofolate reductase; ANC- absolute neutrophils count; WBC, white blood cell; Hb, hemoglobin; PLT, platelet count; i.v., intravenously; i.m, intramuscularly; Cr, Creatinine. TEN, toxic epidermal necrolysis; ARDS, acute respiratory distress syndrome; CRRT, continuous renal replacement therapy*.

* *The day of MTX initiation was D0*.

** *Laboratory tests were not performed*.

Searching for the cause of fever and rashes may be difficult due to their nonspecific characteristics. We also found that the primary toxic effects of LD-MTX may only be limited to nausea, vomiting, and diarrhea, which are common adverse reactions after chemotherapy and are often ignored. However, a persistent fever, progressive skin lesions, and mucositis appeared very soon. The laboratory results showed myelosuppression, with or without damage to the function of the liver and kidney. Myelosuppression is one of the most severe adverse toxic effects of MTX (25). Once severe myelosuppression occurs, secondary infection may lead to sepsis. In the eight cases we summarized, all the patients had three to four degrees of myelosuppression, two patients had sepsis, and one of the two patients died. In this case, myelosuppression mainly manifested as leukopenia and thrombocytopenia, and hemoglobin also has a certain effect. In the majority of cases, the maximum reduction in the number of leukocytes and platelets was estimated to occur approximately 10 days after the last administration of MTX, and the count recovery achieved in the period of 14–21 days (22). In addition, the toxic reaction may cause damage to the cardiopulmonary function and the central nervous system, which is therefore extremely harmful and can be fatal.

Confusingly, the patient was a middle-aged woman and she had no renal insufficiency and wrong administration of LD-MTX, so severe adverse toxic effects were considered as unexpected conditions. To investigate further, we searched for the serum concentration of MTX in time. The laboratory results showed that the MTX concentrations were higher than the aforementioned thresholds. In general, a sustained elevation of plasma MTX concentrations at 24 h (>5–10 μM), 48 h (>1.0 μM), and 72 h (>0.1 μM) after administration of MTX are predictive for the development of toxicity (26). Simultaneously, the personalized genetic testing confirmed that *MTHFR* TT (677C>T) and *MTHFR* AA (1,298A>C) were detected. Therefore, these results confirmed our conjecture, the patient could not tolerate the LD-MTX because her *MTHFR* gene had the polymorphisms mutation.

A previous study reported that the serious adverse toxic effects of LD-MTX may closely be associated with the polymorphisms of *MTHFR* gene (16). *MTHFR* is an important enzyme in the metabolism of folic acid, and is crucial for reproductive function thereby decreasing MTX clearance, as well as increasing the concentration and metabolic accumulation of MTX (15). Two of the most investigated polymorphisms within the *MTHFR* gene are single nucleotide polymorphisms (SNPs) at the mRNA positions 677 (rs1801133) and 1,298 (rs1801131) (8). However, these findings were mainly found in the patients with rheumatoid arthritis or the hematological malignancies (14–16, 27). This is the first report on serious adverse toxic effects of LD-MTX on an EP patient with *MTHFR* mutations. Although gene testing is not usually used as a routine examination before MTX administration, patients with MTX concentration higher than normal and severe adverse toxic effects after LD-MTX treatment should be alert to the possibility of *MTHFR* gene abnormalities.

In this case, individualized and active treatments made the patient achieve a positive outcome. Once the toxicity of LD-MTX is recognized, e.g., high-dose MTX, it is essential to take a series of measures, including CF rescue, adequate hydration, urine alkalinization, monitoring of MTX concentration, and timely treatment of adverse reactions (28). Leucovorin supplies the active form of folic acid, bypassing DHFR inhibition (6). In general, the recommended dose of CF rescue is 12–15 mg/m^2^, with an intravenous drip every 6 h, until the MTX concentration falls below 0.01 uM/L (28, 29). In this case, the serum MTX concentration was still higher than the aforementioned threshold after 4 days of CF rescue with 20 mg, IV q6h, thus, the dose of CF was elevated to 100 mg, IV q6h. However, Shiver et al. pointed that serum MTX concentration is a poor indicator of intracellular toxicity, because MTX is retained in toxic amounts in the polyglutamated form within the cells (6, 19). The status of the patient investigated in this report also confirmed the mentioned finding, in which rescue measures should be taken independent of serum MTX concentration. Besides, reports of removing MTX through hemodialysis and plasmapheresis have mainly concentrated on individual cases, and the efficacy of MTX is still controversial (9, 10, 26). Our patient was given active LD-MTX treatment, in addition to leucovorin rescue, urine alkalinization, intravenous hydration, CRRT, G-CSF, TPO, and blood products, which eventually led to achieve a positive outcome. At the same time, other symptomatic supportive treatments, such as protective isolation, anti-infection treatment, liver protection, and oral and perineal care were given. With the decrease of MTX concentration in blood, the patient was relieved gradually. There was no hypoproteinemia, skin infection, pulmonary infection, acute respiratory failure, acute renal failure, or other fatal complications.

## Conclusion

This is the first report on serious adverse toxic effects of LD-MTX on an EP patient with MTHFR mutations. We aim to alert obstetricians and gynecologists of this rare condition. The unexpected life-threatening toxicity with LD-MTX should be highly considered and early recognized. In particular, some easily overlooked gastrointestinal, skin, and mucosal symptoms occur earlier than severe myelosuppression. When toxic effects are suspected, detecting the polymorphisms of MTHFR gene and monitoring MTX concentration in the blood could assist us to formulate individualized and active treatments.

## Data Availability Statement

The original contributions presented in the study are included in the article/Supplementary Material, further inquiries can be directed to the corresponding author.

## Ethics Statement

The studies involving human participants were reviewed and approved by Medical Ethics Committee of the China Japan Friendship Hospital. The patients/participants provided their written informed consent to participate in this study. Written informed consent was obtained from the individual(s) for the publication of any potentially identifiable images or data included in this article.

## Author Contributions

HY and WW wrote the manuscript. HL, KW, and BL edited and revised the manuscript. All authors contributed to the article and approved the submitted version.

## Funding

This work was supported by the Talent Introduction Program of China-Japan Friendship Hospital, China (2017-RC-4).

## Conflict of Interest

The authors declare that the research was conducted in the absence of any commercial or financial relationships that could be construed as a potential conflict of interest.

## Publisher's Note

All claims expressed in this article are solely those of the authors and do not necessarily represent those of their affiliated organizations, or those of the publisher, the editors and the reviewers. Any product that may be evaluated in this article, or claim that may be made by its manufacturer, is not guaranteed or endorsed by the publisher.
